# Radiation-Induced Carcinogenesis: Mechanistically Based Differences between Gamma-Rays and Neutrons, and Interactions with DMBA

**DOI:** 10.1371/journal.pone.0028559

**Published:** 2011-12-14

**Authors:** Igor Shuryak, David J. Brenner, Robert L. Ullrich

**Affiliations:** 1 Center for Radiological Research, Columbia University Medical Center, New York, New York, United States of America; 2 Department of Radiation Oncology, The University of Texas Medical Branch, Galveston, Texas, United States of America; National Cancer Institute, United States of America

## Abstract

Different types of ionizing radiation produce different dependences of cancer risk on radiation dose/dose rate. Sparsely ionizing radiation (e.g. γ-rays) generally produces linear or upwardly curving dose responses at low doses, and the risk decreases when the dose rate is reduced (direct dose rate effect). Densely ionizing radiation (e.g. neutrons) often produces downwardly curving dose responses, where the risk initially grows with dose, but eventually stabilizes or decreases. When the dose rate is reduced, the risk increases (inverse dose rate effect). These qualitative differences suggest qualitative differences in carcinogenesis mechanisms. We hypothesize that the dominant mechanism for induction of many solid cancers by sparsely ionizing radiation is initiation of stem cells to a pre-malignant state, but for densely ionizing radiation the dominant mechanism is radiation-bystander-effect mediated promotion of already pre-malignant cell clone growth. Here we present a mathematical model based on these assumptions and test it using data on the incidence of dysplastic growths and tumors in the mammary glands of mice exposed to high or low dose rates of γ-rays and neutrons, either with or without pre-treatment with the chemical carcinogen 7,12-dimethylbenz-alpha-anthracene (DMBA). The model provides a mechanistic and quantitative explanation which is consistent with the data and may provide useful insight into human carcinogenesis.

## Introduction

Experimental investigation of the induction of pre-malignant growths and tumors in laboratory animals exposed to various carcinogens is a well-established method for gaining insight into the mechanisms of carcinogenesis, from which better understanding of cancer in humans can be achieved [1], [2]. In particular, different types of ionizing radiation produce different dependences of cancer risk on radiation dose and dose rate. For radiation doses which are sufficiently low to avoid substantial cell killing, sparsely ionizing radiation, such as γ-rays, generally produces dose response shapes which are linear or with a positive second derivative, e.g. upwardly curving linear-quadratic functions. When the dose rate is reduced, i.e. the same dose is delivered over a longer time, the risk generally decreases and the dose response becomes a linear function of dose [3], [4], [5]. This is called a direct dose rate effect. In contrast, densely ionizing radiations, such as neutrons and α-particles, tend to produce dose response shapes with a negative second derivative, i.e. downwardly curving, where the risk initially grows with dose, but eventually reaches a plateau and/or begins to decrease. When the dose rate is reduced, the risk generally increases [6], [7], [8], [9], [10]. This is called an inverse dose rate effect.

These qualitative differences in dose response shape and dose rate dependences suggest qualitative differences in the mechanisms of carcinogenesis by sparsely and densely ionizing radiation. Both radiation types produce ionizing tracks, which consist of the highly correlated ionizations and excitations produced by a single incident photon, electron, neutron, or other particle within a very short time. An ionizing track can damage biomolecules, e.g. produce double strand breaks in DNA [11], [12]. Damage can be generated by each track which traverses a cell, and damage produced by two (or more) different ionizing tracks can interact. If such damage is not repaired correctly, it has the potential to initiate the cell into a pre-malignant state, placing it on the path towards eventual tumor formation.

The yield of incorrectly repaired damage from one-track action is generally proportional to the number of tracks traversing a cell, i.e. to the radiation dose. The damage yield from two-track action (i.e. due to interactions of damage generated by two independent tracks) is generally nonlinear (often quadratic) in dose. When the radiation dose is delivered over a longer time period (i.e. when the dose rate is reduced), traversals of the cell by ionizing tracks occur farther apart in time. This allows for damage repair during irradiation and reduces the probability that damage induced by a given track will interact with damage induced by a subsequent track [12], [13]. Consequently, one-track damage typically results in linear dose responses with no dependence on dose rate, and two-track damage typically results in quadratic dose responses which are reduced in magnitude by reducing the dose rate – i.e. a direct dose rate effect.

A track of densely ionizing radiation is generally much more damaging to a cell than a track of sparsely ionizing radiation [11], [14]. Traversal by multiple tracks of sparsely ionizing radiation is often required for accumulation of damage sufficient to initiate (or kill) a cell. In contrast, initiation or clonogenic death can be produced by only a few tracks (or even a single track) of densely ionizing radiation [14]. Consequently, interaction of damage from multiple tracks contributes substantially to carcinogenesis induced by sparsely ionizing radiation, whereas densely ionizing radiation induces cancers mainly through single-track action.

Because cells in a tissue communicate with each other through a variety of signals, those cells which have not themselves been traversed by ionizing tracks, but have received signals from cells which have been traversed, can experience non-targeted effects of radiation called radiation-bystander effects. Such effects include altered differentiation, proliferation and migration, altered redox balance and gene expression, cell death (e.g. apoptosis), as well as various forms of genomic damage (e.g. micronuclei, mutagenesis, chromosome damage) [15], [16], [17], [18], [19], [20], [21], [22], [23], [24], [25]. We have suggested [7], [26], [27] that the radiation-bystander effect is important for carcinogenesis induced by densely ionizing radiation and may be (at least partially) responsible for the shape of the dose response and for observed inverse dose-rate effects. In brief, we assumed [27] that when a cell is traversed by an ionizing track, it has some probability of being moved into an “activated” state, e.g. a state of oxidative stress. Intercellular signals can propagate to surrounding cells up to a considerable distance, and cause some of those bystander cells, which have not been irradiated, to also become activated. Eventually, the activated state reverts back to the background state.

Because cell activation is a binary (“on” or “off”) phenomenon, increasing the number of closely-timed ionizing track traversals per cell beyond the number needed for activation will not increase the activation probability, or the intensity of signals released to surrounding cells. This explains saturation of bystander responses as radiation dose is increased [17], [28], [29], which leads to downwardly curving dose response curves (i.e. those with a negative second derivative). Such dose response shapes tend to produce inverse dose rate effects. This can be demonstrated by mimicking a decrease in dose rate by splitting the dose into two equal fractions [7], [26]: If the dose response is linear (i.e. its second derivative is zero), the sum of the responses to both fractions would be the same as the response to the total dose – no dose rate effect. If the dose response is upwardly curving (i.e. its second derivative is positive), the sum of the responses to both fractions would be smaller than the response to the total dose – a direct dose rate effect. However, if the dose response is downwardly-curving (i.e. its second derivative is negative), the sum of the responses to both fractions would be greater than the response to the total dose – an inverse dose rate effect.

As an example, suppose that one ionizing track traversal is sufficient to activate a cell. If several track traversals occur within a short time, the radiation-bystander effect will be the same as for one traversal. However, if the dose rate is reduced so that the average time between traversals becomes longer than the average time needed for an activated cell to return back to the background state, the duration of cell activation (and, hence, of the radiation-bystander effect) will be prolonged, i.e. an inverse dose rate effect will occur.

We hypothesize that the dominant mechanism for induction of many solid cancers by sparsely ionizing radiation is direct damage to stem cells, resulting in initiation of these cells to a pre-malignant state, producing new (radiation-induced) pre-malignant cell clones. Such clones can subsequently expand in cell number by proliferation (promotion), and some cells within them can acquire additional mutations to become fully malignant (transformation). Finally, some of the transformed cells can eventually develop into tumors (progression) [30], [31], [32]. In contrast, we hypothesize that for densely ionizing radiation the dominant carcinogenesis mechanism is indirect (radiation-bystander-effect mediated) promotion of the growth of already existing pre-malignant cell clones. Such clones were initiated by spontaneous processes, and/or exposure to other agents (e.g. DMBA) that may initiate additional clones which may not result in a substantially increased cancer risk on their own, but can become activated by radiation-bystander signals during irradiation. Activation can cause these clones to increase in size, e.g. due to elevated proliferation rates, decreased death/differentiation rates, and disrupted intercellular signaling. This process results in radiation-bystander effect-mediated promotion of the growth of pre-malignant cell clones, increasing the subsequent risk of cancer at low doses, particularly when delivered at low dose rates.

Here we present a mathematical model based on these assumptions and test it using data on mammary carcinogenesis (incidence of dysplastic growths and tumors in the mammary glands) in mice exposed to high or low dose rates of γ-rays and neutrons, either with or without pretreatment with a low initiating dose of the chemical carcinogen 7,12-dimethylbenz-alpha-anthracene (DMBA). DMBA is metabolized into reactive compounds which can produce potentially mutagenic and carcinogenic DNA adducts [33], [34], [35], and ionizing radiation can damage DNA in multiple ways, most importantly by the production of double strand breaks (DSBs) [11], [12], [36], [37]. Mechanisms similar to those for mammary cancer may apply to other solid cancers, such as those of the lung [38], [39], [40]. In contrast, leukemia may be driven more by induction of specific radiation-induced cytogenetic effects [41], [42].

## Results

**Figure 1:**
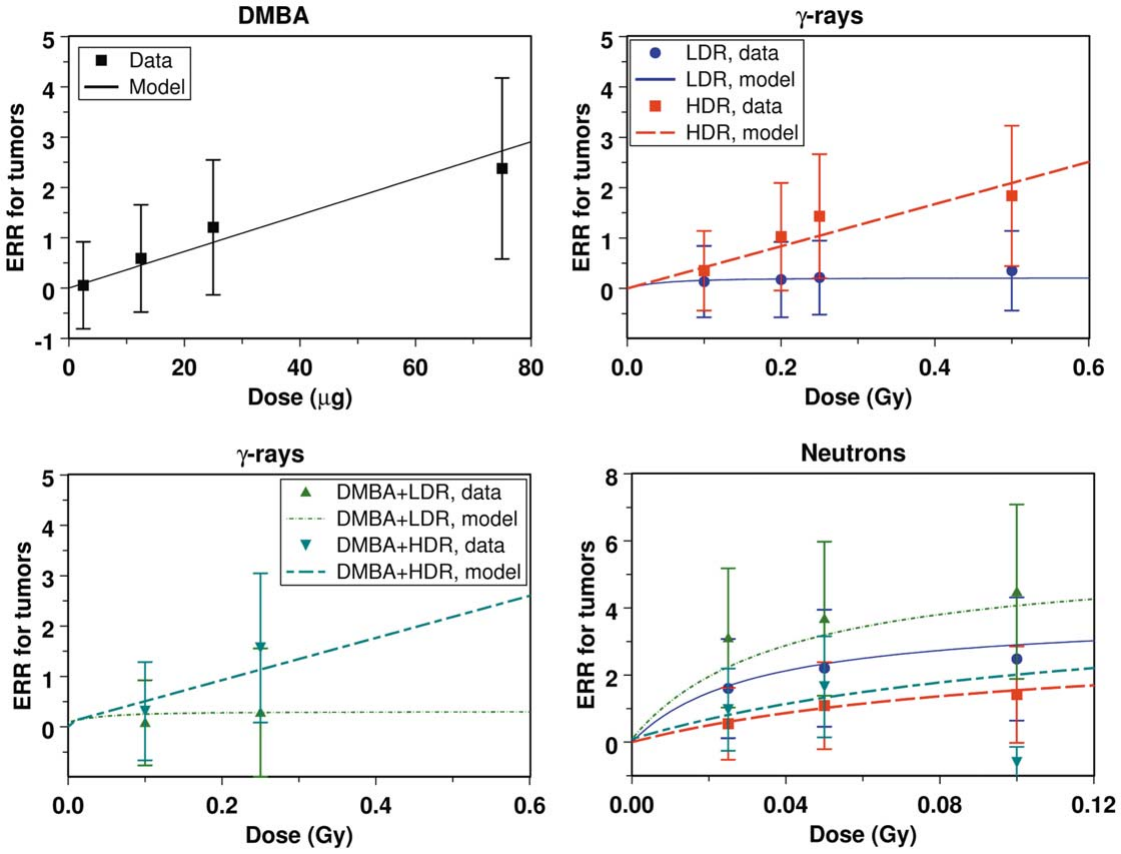
The data and model predictions for mammary tumor ERR after γ-ray, neutron and/or DMBA exposure. In the panels showing effects of γ-rays and neutrons, the legend is the same: HDR = high dose rate; LDR = low dose rate; DMBA = 2.5 µg of DMBA. In this and the following figures error bars represent 95% confidence intervals.

**Figure 2:**
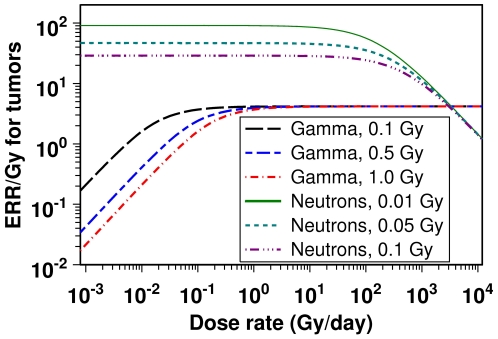
Model predictions for tumor ERR/Gy as function of dose/dose rate for γ-rays and neutrons.

The mechanistic mathematical model developed here is consistent with the data for mouse mammary tumor and dysplasia induction by three different carcinogens (γ-rays, neutrons, and DMBA), administered at different doses and dose rates (Figs. 1–2). Notably, a single set of parameters was used for all the data. Best-fit parameter values and parameter meanings are presented in Table 1.

**Table 1:** Best-fit model parameters for the excess relative risk (ERR) of mammary tumors and dysplasias in mice treated with DMBA and/or irradiated with γ-rays and neutrons at high or low dose rates.

Parameter	Units	Best-fit value	95% confidence interval
*b*	days^−1^	1.40×10^−2^	0.42×10^−2^, 2.3×10^−2^
*δ*	days^−1^	4.00×10^−4^	2.1×10^−4^, 5.7×10^−4^
*X_DMBA_*	days/µg	8.42	7.3, 11
*X_g_*	days/Gy	969	870, 1300
*K_rep_*	days^−1^	0.391	0.36, 0.42
*Y_n_*	days^−1^	2.51×10^4^	2.0×10^4^, 3.8×10^4^
*q*	Gy/day	123	110, 150
*L*	days	50	–

Parameter interpretations are: *b* = pre-malignant niche replication rate; *δ* = the parameter for homeostatic regulation of the number of pre-malignant cells per niche; *X_DMBA_* and *X_g_* = cell initiation constants for DMBA and γ-rays; *K_rep_* = constant for repair of γ-ray-induced cell-initiating damage; *Y_n_* = neutron-induced bystander promotion constant; *q* = radiation dose rate at which 50% of all susceptible cells are activated by radiation-bystander signals under steady-state conditions; *L* = lag time between the appearance of the first malignant cell and tumor diagnosis.

### Tumors

The data for tumor induction by DMBA are consistent with a linear dose response (Fig. 1) generated by the assumption that DMBA is an initiating agent. As suggested by the value of the DMBA-induced initiation parameter *X_DMBA_* (Table 1), each µg of this carcinogen is approximately as efficient at initiating mammary tumors as 8 days of spontaneous initiation. The γ-ray data are also consistent with the interpretation that this radiation type acts as an initiating agent, with a linear dose response at high dose rates. As suggested by the value of parameter *X_g_*, one Gy of high dose rate γ-radiation is approximately as efficient at initiating mammary tumors as 1000 days of spontaneous initiation (Table 1). At lower dose rates this efficiency is reduced due to repair of radiogenic damage (which can cause initiation) during exposure. This direct dose rate effect for γ-rays can be seen in Fig. 1 and Fig. 2. For example, when the dose rate is reduced to 0.01 Gy/day, the tumor ERR is reduced by approximately an order of magnitude compared with the highest tested dose rate of 576 Gy/day. Pre-treatment of γ-irradiated mice with 2.5 µg of DMBA has little effect on increasing the predicted tumor ERR because initiating effects are assumed to be additive, as discussed in more detail below. This is consistent with the data (Fig. 1). Of course, the model fits to the data do not rule out promoting effects of DMBA or γ-rays, but such effects are probably not dominant.

**Figure 3:**
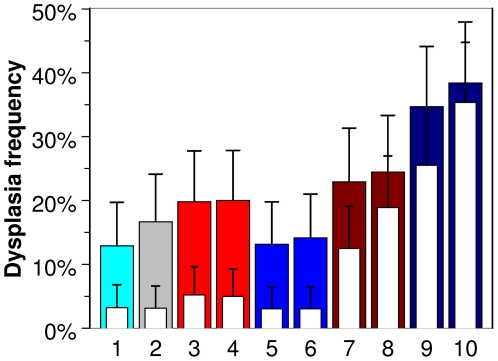
Frequencies of ductal mammary dysplasias observed in the fat pads of untreated mice after transplant of mammary epithelial cells from carcinogen-treated mice. 1 = control group, 2 = treated with 2.5 µg DMBA, 3 = 0.25 Gy γ-rays at high dose rate, 4 = 2.5 µg DMBA+0.25 Gy γ-rays at high dose rate, 5 = 0.25 Gy γ-rays at low dose rate, 6 = 2.5 µg DMBA+0.25 Gy γ-rays at low dose rate, 7 = 0.025 Gy neutrons at high dose rate, 8 = 2.5 µg DMBA+0.025 Gy neutrons at high dose rate, 9 = 0.025 Gy neutrons at low dose rate, 10 = 2.5 µg DMBA+0.025 Gy neutrons at low dose rate. Filled bars = total dysplasias (measured 10 weeks after transplant), empty bars = persistent dysplasias (those visible 16 weeks after transplant). Details are discussed in the main text.

For neutrons, however, the data are consistent with the interpretation that this type of radiation acts mainly by radiation-bystander effect mediated promotion, rather than by initiation. Such an interpretation explains the following patterns exhibited by the neutron data: (1) An inverse dose rate effect (Figs. 1 and 3). (2) A dose response shape with a negative second derivative (Fig. 1). (3) Synergistic (rather than additive) interactions between neutrons and 2.5 µg of DMBA (Fig. 1), which are predicted to result from promotion by neutrons of cells/clones which have been initiated by DMBA. These issues are discussed in more detail below.

### Dysplasias

Whereas tumors represent the final endpoint in the mammary carcinogenic process, it was also possible to measure the incidence of earlier-stage pre-malignant lesions following exposure to a low initiating dose of 2.5 µg of DMBA, neutrons, or γ-rays with or without DMBA exposure one week prior to irradiation. These studies used a model system that takes advantage of the fact that mammary fat pads of 3-week-old mice from which the rudimentary mammary tissue is removed and which, therefore, contain no mammary cells of their own, serve as an ideal site for the growth and differentiation of mammary cells into functional mammary glands. As shown previously, pre-malignant cells can be quantified by their expression as ductal dysplasias in mammary outgrowths derived from carcinogen-treated mammary epithelium [43], [44]. Although the premalignant nature of these cells was found to be a stable characteristic, the expression of their premalignant state was found to be a function of the growth state of the mammary outgrowth. During active growth and development (8–10 weeks following injection of cells into the fat pad) the frequency of dysplastic outgrowth was higher than when the outgrowth was mature (16 weeks post injection). These results indicated that expression of the premalignant phenotype is regulated through interactions with the microenvironment. Acquisition of the potential for autonomous growth by these pre-malignant cells can be quantified by determining the frequency of persistent lesions 16 weeks post-injection. As a result, both the induction of premalignant cells (as total dysplasias measured 10 weeks post-injection), and their subsequent dynamics (as persistent dysplasias at 16 weeks post injection) can be quantified.

Results are shown in Fig. 3. Exposure to DMBA at a dose of 2.5 µg, which does not result in a substantial increase in the frequency of mammary tumors (based on the best-fit parameter values in Table 1, 2.5 µg of DMBA is approximately equivalent to 20 days of spontaneous initiation, whereas 1 Gy of γ-rays is approximately equivalent to 1000 days), increased the frequency of premalignant cells, but the large majority of these cells did not acquire the potential for autonomous growth and the dysplastic growths regressed. Similarly, exposure to γ-rays with or without prior exposure to DMBA resulted in an increase in premalignant cells, but only a small fraction of these cells persisted. These results are consistent with γ-rays and DMBA as initiators with very little, if any, impact on the promotion of initiated cells. In contrast, exposure to a very low dose of neutrons increased both the frequency and persistence of premalignant cells. This was particularly dramatic following low dose rate exposures. Given the low total dose (0.025 Gy) which would result in only a fraction of the mammary cells being directly hit, these data support a significant role for the radiation-bystander effect (which, as our analysis suggests, acts mainly through promotion of pre-malignant cell clone growth) in the neutron-induced carcinogenic process.

**Figure 4:**
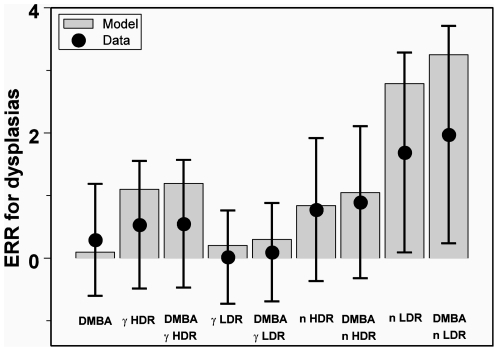
The data and model predictions for dysplasia ERR after exposure to γ-rays, neutrons and/or DMBA. Legend: DMBA = 2.5 µg of DMBA; γ = 0.25 Gy of γ-rays; n = 0.025 Gy of neutrons; HDR = high dose rate; LDR = low dose rate.

Because the experimental procedure for measuring dysplasia incidence was different from the procedure for measuring tumor incidence (in the former case, cells from carcinogen-treated animals were transplanted into untreated animals, whereas in the latter case there was no such manipulation of the mammary glands of treated animals), it is not surprising that there are some systematic differences in the magnitudes of the dose responses for tumors and dysplasias. In general, the ERRs for dysplasias were approximately a factor of 1.5–2.0 lower than the ERRs for tumors (see Figs. 1 and 4). We felt that this difference is not large enough to warrant introducing a different parameter set for dysplasias, and the ERR data for both tumors and dysplasias were fitted together by the model using a single parameter set presented in Table 1. Consequently, the best-fit predicted ERR values for dysplasias tended to be somewhat higher than the data points, due to influence from the tumor ERRs, but this difference did not exceed the 95% confidence intervals of the data points (Fig. 4).

### Model fit

Goodness of fit of the model was estimated by the reduced X^2^ method, which is defined as X^2^ divided by the degrees of freedom. A good fit is suggested by reduced X^2^ near 1, whereas values >1 suggest a poor fit and values <1 suggest overfitting. Using the default model (Eqs. 3–4 in the Materials and Methods section), which assumes that DMBA and γ-rays are pure initiators and neutrons are a pure promoter, the reduced X^2^ was 1.35. If γ-rays were assumed to act as a promoter rather than as an initiator (i.e. extending the neutron model to γ-ray data), the reduced X^2^ increased to 1.51. If neutrons were assumed to act as an initiator rather than as a promoter (i.e. extending the γ-ray model to neutron data), the reduced X^2^ increased even more to 2.55.

## Discussion

Our analysis suggests that the neutron-induced risks of mammary carcinogenesis for the selected mouse strain are dominated by radiation-bystander effect-mediated promotion of already pre-malignant cell clones. Initiation by neutrons of normal cells into the pre-malignant state could not be ruled out, but was not necessary for explaining the data. Modulation of the promoting effects of radiation due to homeostatic regulation of the average number of stem cells per niche (determined by parameter *δ*) was found to be important. This parameter was responsible for preventing niches filled with pre-malignant cell clones from becoming very large during irradiation by opposing the proliferative promotion process. Consequently, the neutron dose response shape became “flattened”, i.e. had a negative second derivative (Fig. 1).

Over time since exposure, a value of *δ*>0 predicts that pre-malignant niche sizes are gradually reduced towards the default sizes set before irradiation, thereby reducing the ERR. This suggests that homeostatic mechanisms in the organ oppose the accumulation of dysplastic pre-malignant cells resulting in a reduction in their number and/or carcinogeneic potential over time after exposure. Such an interpretation is consistent with the experimental data on pre-malignant mammary dysplasias: over time since exposure many dysplastic lesions regress (Fig. 3). This does not mean that those lesions which can no longer be observed have been eliminated completely (in fact the persistence of such occult initiated cells has been directly demonstrated previously using the dysplasia model system [44]), but the number of pre-malignant cells per lesion has probably been reduced. The promotional effect of low dose rate neutron exposure opposes the homeostatic mechanisms, causing the proportion of dysplasias which persist to increase (Fig. 3).

The explanation for neutron-induced mammary tumor risk suggested by our model, which is based on radiation-bystander effect-mediated promotion, also provides a description for the inverse dose rate effect seen clearly in the analyzed data (i.e. that tumor risk per unit dose is higher at dose rates of 0.01 Gy/day compared with higher dose rates). In essence, in a radiation-bystander promotion-driven model the total duration of irradiation, which varies inversely with dose rate, is the major determinant of risk. Qualitatively, the conclusions drawn from this analysis are the same as those presented previously in analyses of radon-induced lung cancer risks in rats and humans [38], [39], [40], possibly because densely ionizing radiation such as α-particles emitted by radon and its progeny and neutrons may have qualitatively similar biological effects.

In contrast with the dose responses seen with neutron exposure, γ-rays in the same mouse strain produce a relatively linear dose response in the dose range up to 0.5 Gy. Instead of an inverse dose rate effect, a direct dose rate effect is seen, as expected for sparsely ionizing radiation. As mentioned previously, these differences in the data suggest that the carcinogenesis mechanisms are very different for neutrons vs. γ-rays: the radiation-bystander effect is probably very important for the former, and not (or less) important for the latter.

When DMBA and γ-rays are combined, additive effects from these initiators are expected. However, when DMBA and neutrons are combined, a synergistic interaction is expected because neutrons can promote the growth of pre-malignant clones initiated by DMBA. This is consistent with the data for both tumors (Fig. 1) and dysplasias (Fig. 3). For example, the combination of 2.5 µg DMBA and low dose rate neutrons produced substantially higher tumor ERRs than the same doses of low dose rate neutrons alone (Fig. 1): at 0.025 Gy of low dose rate neutrons the tumor ERR was 1.6 without DMBA and 3.1 with DMBA, at 0.05 Gy it was 2.2 and 3.7, and at 0.1 Gy it was 2.5 and 4.5, respectively. For high dose rate neutrons, where the promoting effect per given dose would be reduced because of reduced duration of exposure, the interaction of neutrons with DMBA was less noticeable: at 0.025 Gy of high dose rate neutrons the tumor ERR was 0.5 without DMBA and 1.0 with DMBA, at 0.05 Gy it was 1.1 and 1.6, and at 0.1 Gy it was 1.4 and -0.7, respectively. Error bars for these estimates are shown in Fig. 1. A qualitatively similar pattern was observed for the frequency of persistent dysplasias (those present at 16 weeks post-injection). At 0.025 Gy of low dose rate neutrons the persistent dysplasia frequency was 25.5% without DMBA and 35.4% with 2.5 µg DMBA, and for high dose rate neutrons the respective frequencies were 12.5% and 18.9%. Error bars are shown in Fig. 3. For γ-rays, at either high or low dose rate, combination with DMBA had only small effects on tumor ERR or dysplasia frequency (Figs. 1 and 3).

In summary, the analysis presented here provided a mechanistic quantitative model based on the hypothesis that carcinogens such as DMBA and γ-rays act mainly as initiators of pre-malignant cells, whereas neutrons act mainly to promote the growth of already existing pre-malignant cell clones. The model was then directly tested using data for mammary carcinogenesis as well as early events in the carcinogenic process induced by DMBA, γ-rays and neutrons in a well-established mouse mammary cancer animal model. Importantly, the results support other evidence [38], [39], [40] suggesting that densely ionizing radiation, such as neutrons and α-particles, induces cancer mainly through promoting effects on pre-malignant cell clones. Such promoting effects may occur due to disruption of intercellular signaling by radiation, which can happen even if only a small fraction of the cells are actually traversed by ionizing tracks.

## Materials and Methods

### Model used

We previously assumed [27] that a susceptible cell (e.g. a mammary stem cell) will have a probability *P_a_* of being in the activated bystander state, rather than in the background bystander state, with *P_a_* described by the following differential equation:
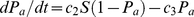
(1)Here *S* is the average concentration of the bystander signal(s) in the target organ, *c_2_* determines cell activation (units = time^−1^×concentration^−1^), and *c_3_* determines the opposite process, i.e. cell deactivation back to the background state (units = time^−1^). The term *c_2_ S* represents our assumption that the probability per unit time of converting a cell from the background state to the activated state is proportional to the concentration of the bystander signal. The term 1−*P*_a_ indicates the probability that the given cell has not yet undergone this transition and that it is only such cells that are available for being activated. This term thus describes the “saturation” phenomenon characteristic of radiation-bystander responses (e.g. [28], [29]). Finally, the term −*c_3_ P*_a_ represents the assumption that the activated state tends to spontaneously revert to background at a fixed probability per unit time.

In the present paper we again use Eq. 1. We assume here that the time-average over a short time of the signal concentration, *S*, rapidly reaches a steady-state value in the target organ [45], which is linearly proportional to the radiation dose rate, *R*. This assumption can be derived from the plausible supposition that each cell traversed by an ionizing track sends out the bystander signal briefly. During protracted irradiation, cells throughout the organ are hit at random continually. Thus, at any location the time average of *S* over a characteristic relaxation time *T_c_* (i.e. the time needed to revert from the activated state to the background state, *T_c_* = 1/*c_3_*) is spatially and time independent (after the initial and before the final transients, which are assumed to be short), and is proportional to *R*.

This assumption would be less likely to hold at very high dose rates, where most or all cells are traversed by ionizing tracks at least once per relaxation time (*T_c_*). However, at such high dose rates, essentially all cells susceptible to activation by bystander signals would be activated (*P*_a_ = 1), so any deviation of the dependence of signal concentration on dose rate from linearity (e.g. saturation of signal production) would not affect model predictions. At low dose rates, where each individual cell would be expected to be traversed at most once per relaxation time, differences in dose rate would not matter to this particular cell. However, the group of many cells communicating through bystander signaling, which is the radiation “target” under our assumptions, would be traversed by several tracks per relaxation time even at quite low dose rates, so dependence of the group response on dose rate is expected.

The assumed linear dependence of the steady-state bystander signal concentration on radiation dose rate can be represented mathematically by the function *S* = ρ *R*, where ρ is a proportionality constant (units = time×concentration×dose^−1^). Defining a new parameter *k_1_* = *c_2_* ρ (units = dose^−1^), Eq. 1 can thus be rewritten as follows: *dP*_a_/*dt* = *c_2_* ρ *R* (1−*P*_a_)−*c_3_ P*_a_ = *k_1_ R* (1−*P*_a_)−*c_3_ P*_a_. An analytic solution for this expression is available. However, to simplify the final model, it is possible to approximate by using the equilibrium condition *dP_a_*/*dt* = 0, and solving for *P_a_*. To reduce the number of adjustable parameters, we introduce a new parameter *q* (* = c_3_/k_1_*, units = dose×time^−1^), interpreted as the radiation dose rate at which 50% of all susceptible cells are activated under steady-state conditions. Given these assumptions and manipulations, the equilibrium value of *P_a_* is:
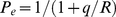
(2)


The adjustable parameter *q* is determined by multiple factors such as bystander signal range (which affects *k_1_*) and cell turnover rate (which affects *c_3_*). If *q* is small, *P_e_* approaches saturation (unity) at lower dose rates than when *q* is large. Under background conditions without excess radiation exposure, *P_e_* = 0.

The assumptions and mathematical implementation of our carcinogenesis model were described in detail in previous papers [46], [47]. Briefly, the model uses a two-stage approach similar to the classic two-stage clonal expansion model [8], [48], [49], where normal stem cells can be initiated into a pre-malignant state, either spontaneously or by radiation. A surviving initiated cell can grow into a pre-malignant clone, which quickly takes over the stem cell niche [50], [51], [52], [53], [54], [55], [56], [57] or tissue compartment (e.g. a colonic crypt) in which it originated. Niche boundaries initially constrain further growth of the clone, so once the initial niche is filled, clonal expansion proceeds by a much slower process of pre-malignant niche replication or invasion and takeover of adjacent normal niches by pre-malignant cells [57], [58], [59], [60], [61]. This slow process occurs on the scale of multiple years and decades in humans (and months in mice), and may involve acquisition of new mutations [62]. Each pre-malignant cell in any niche has some probability of transforming into a fully malignant cell and, after some lag time (*L*), into a tumor [8], [48], [49]. The per-cell probability of malignant transformation decreases with the age of the individual, e.g. due to reduced stem cell proliferation and self-renewal rates, reduced background malignant transformation rates, and/or elevated death rates [63], [64], [65], [66], [67].

The mathematical expression for the excess relative risk (ERR) of cancer following irradiation was derived previously: see Eq. 15 in [46]. For a single radiation exposure which occurs in a sufficiently short time for cell proliferation to be neglected, this expression can be simplified: see Eq. 2 in [68]. If the radiation dose is sufficiently small for cell killing to be neglected, as can be done for the experimental data analyzed here where the maximum γ-ray dose was 0.5 Gy and the maximum neutron dose was 0.1 Gy, further simplification is possible and the ERR is given by:
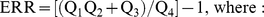
(3)

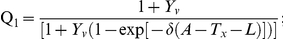


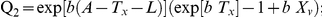


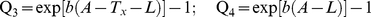
Here *A* is the age, *T_x_* is the time at exposure, *L* is the lag time between the appearance of the first malignant cell and tumor diagnosis, *b* is pre-malignant niche replication rate (units = time^−1^), *δ* is the parameter for homeostatic regulation of the number of pre-malignant cells per niche (units = time^−1^), *X_v_* is the term for initiation of new pre-malignant cells/clones (units = time), and *Y_v_* is the term for promotion of already pre-malignant cell clones (units = dimensionless).

The functions *X_v_* and *Y_v_* are given by the following expressions:
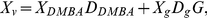
(4)

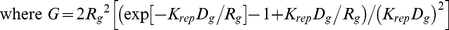


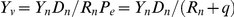
Here *D_DMBA_*, *D_g_* and *D_n_* are the doses for DMBA, γ-rays and neutrons, respectively, *R_g_* and *R_n_* are the dose rates, *X_DMBA_* and *X_g_* are cell initiation constants for DMBA and γ-rays (units = time×dose^−1^), *K_rep_* is the constant for repair of γ-ray-induced cell-initiating damage (units = time^−1^), *Y_n_* is the neutron-induced bystander promotion constant (units = time^−1^), and *q* is the radiation dose rate at which 50% of all susceptible cells are activated by radiation-bystander signals under steady-state conditions. *G* is the Lea-Catcheside factor for repair of radiation-induced damage for exposures at constant dose rate [69]. This functional form is very commonly used to describe radiation protraction and fractionation effects. *P_e_* is the equilibrium value of the cell activation probability, taken from Eq. 1. As discussed above, the functional forms for *X_v_* and *Y_v_* reflect our assumptions that: (1) DMBA acts exclusively as an initiating agent; (2) γ-radiation also acts exclusively as an initiating agent, and the initiating damage it causes is subject to repair; (3) neutrons act exclusively as a promoting agent through radiation-bystander effect-mediated mechanisms.

### Data sets and model fitting procedure

The data on mammary tumors and dysplasias in female BALB/C mice were accumulated by the Ullrich laboratory as previously described [43], [44]. For tumor induction, the mice were exposed to the following carcinogens at the age of 12 weeks (84 days): (1) γ-ray doses up to 0.5 Gy, at a low dose rate of 0.01 Gy/day or at a high dose rate up to 0.4 Gy/min (576 Gy/day); (2) fission-spectrum neutron doses up to 0.1 Gy, at a low dose rate of 0.01 Gy/day or at a high dose rate up to 0.25 Gy/min (360 Gy/day); (3) 7,12-dimethylbenz-alpha-anthracene (DMBA) doses up to 75 µg; (4) combinations of 2.5 µg DMBA with high- or low-dose rate γ-rays or neutrons, with the DMBA given 1 week prior to irradiation. These carcinogen doses were not high enough to produce acute lethality in the mice. Mammary tumor incidences were measured up to the age of 800 days. A total of 3775 mice were used in these tumorigenesis experiments, with 285 mice in the control group, 390 mice exposed to DMBA, 1560 exposed to γ-rays, 338 exposed to combinations of DMBA and γ-rays, 590 exposed to neutrons, and 612 exposed to combinations of DMBA and neutrons. Mice were maintained in specific-pathogen-free conditions at the AAALAC-approved Laboratory Animal Resource facility at Colorado State University. All animal work was approved by the Institutional Animal Care and Use Committee at Colorado State University.

For dysplasia induction, mice were irradiated with 0.25 Gy γ-rays (high- or low-dose rate), or 0.025 Gy of neutrons (high- or low-dose rate), and/or treated with 2.5 µg DMBA. Epithelial cells were removed from the mammary glands of irradiated mice 16 weeks after exposure, and transplanted into fat pads of unirradiated recipient mice, which have previously been cleared of mammary tissue. The total number of ductal dysplasias, which is a surrogate for the total number of pre-malignant stem cells, was measured 10 weeks after transplantation, and the number of such dysplasias which persist for longer time was measured 16 weeks after transplantation. A total of 966 mice were used in these dysplasia experiments, with 93 mice in the control group, 96 mice exposed to DMBA, 195 exposed to γ-rays, 199 exposed to combinations of DMBA and γ-rays, 194 exposed to neutrons, and 189 exposed to combinations of DMBA and neutrons.

The measured incidences of mammary tumors and total frequency of dysplasias after each exposure scenario were converted to excess relative risks (ERR). Fitting of the model to the ERRs was carried out using a customized random-restart simulated annealing algorithm implemented in the FORTRAN language, using standard inverse variance weighting. A single parameter set was used for both tumors and dysplasias. The parameters were freely adjustable, with only non-negativity constraints. The delay period between appearance of the first malignant cell and detectable cancer was assumed to be *L* = 50 days, and exploratory calculations showed that the predictions are not very sensitive to this parameter. Ninety-five percent confidence intervals for all adjustable parameters were estimated by generating multiple synthetic data sets based on the experimental data set and fitting the model to these synthetic data sets. The simulated data sets were produced using the data points assuming the normal distribution.
